# Detection of *Aeromonas*, *Campylobacter* and *Salmonella* using concurrent bacterial culture and commercial multiplex PCR, Sydney, Australia, 2023

**DOI:** 10.2807/1560-7917.ES.2026.31.5.2500355

**Published:** 2026-02-05

**Authors:** Christopher Yuwono, Michael C Wehrhahn, Eve Slavich, Fang Liu, Li Zhang

**Affiliations:** 1 School of Biotechnology and Biomolecular Sciences, University of New South Wales, Sydney, Australia; 2 Douglass Hanly Moir Pathology, a Sonic Healthcare Practice, Macquarie Park, Sydney, Australia; 3 Stats Central, Mark Wainwright Analytical Centre, University of New South Wales, Sydney, Australia

**Keywords:** *Aeromonas*, *Campylobacter*, *Salmonella*, gastroenteritis, diarrhoea

## Abstract

**BACKGROUND:**

*Aeromonas, Campylobacter* and *Salmonella* species can cause gastrointestinal infections in humans. Information on the performance of detection methods is relevant to assess the impact of changes on surveillance.

**AIM:**

We aimed to assess detection of *Aeromonas*, *Campylobacter* and *Salmonella* by culture and Seegene multiplex real-time PCR in faecal samples from patients with gastrointestinal symptoms.

**METHODS:**

In 2023, all faecal samples submitted to a clinical microbiology laboratory in Sydney, Australia, were tested for *Aeromonas*, *Campylobacter* and *Salmonella* species by culture and Seegene PCR, both detecting at the genus level. The results were analysed descriptively and with binomial generalised linear model, quantile regression, censored regression model and Wald tests.

**RESULTS:**

Of the 90,291 samples tested, more samples were positive with PCR than with culture for *Aeromonas* (PCR: 2.9%; culture: 0.5%) and *Campylobacter* (PCR: 4.2%; culture: 3.1%), but fewer for *Salmonella* species (PCR: 0.7%; culture: 0.8%). Of the culture-positive samples, 19.2% were negative for *Aeromonas* by PCR, 3.6% for *Campylobacter* and 23.0% for *Salmonella*. Of the PCR-positive samples, 81.9% were negative for *Aeromonas* by culture, 25.6% for *Campylobacter* and 14.2% for *Salmonella*. Quantification cycle (Cq) values were negatively associated with patient age for *Aeromonas,* indicating higher bacterial loads in older patients and positively associated with age for *Campylobacter*, indicating lower bacterial loads in older patients (p < 0.001). Also, Cq values were negatively associated with detection by culture and faecal calprotectin levels (p < 0.001).

**CONCLUSION:**

These findings highlight the importance of pathogen- and method-specific interpretation of PCR and culture results in diagnostic testing and surveillance.

**Key public health message**

**What did you want to address in this study and why?**

*Aeromonas*, *Campylobacter* and *Salmonella* are bacteria that can cause diarrhoeal infections in humans. We wanted to investigate how well two common laboratory methods (culture and PCR) detect these pathogens from stool samples and if detection varies by age and sex. Knowing this helps us understand why some infections may not be diagnosed.

**What have we learnt from this study?**

Of the 90,291 stool samples tested, with PCR, more samples were positive for *Aeromonas* (2.9% vs 0.5%) and *Campylobacter* (4.2% vs 3.1%), while culture detected more *Salmonella* (0.8% vs 0.7%). Detection of the three pathogens varied by patient age: *Aeromonas* was most often detected in samples from patients aged > 60 years, *Campylobacter* in samples from 20–29-year-old and *Salmonella* in 0–4-year-old patients.

**What are the implications of your findings for public health?**

Using both methods may help doctors find more infections. Understanding how age affects predisposition to these pathogens could guide better diagnosis and treatment. Our findings also help the interpretation of diagnostic test results and surveillance data.

## Introduction

*Aeromonas*, *Campylobacter* and *Salmonella* species are important causes of bacterial gastrointestinal infections in humans. *Aeromonas* species are emerging human enteric pathogens, infecting people with normal and weakened immune systems, typically through contaminated food or water [1]. The incidence of *Aeromonas*-related gastrointestinal infections varies by region, with one Australian study reporting *Aeromonas* as the second most common enteric bacterial pathogen [2]. While gastrointestinal infections with *Aeromonas* species most commonly present as gastroenteritis, they can also lead to biliary infections, such as cholangitis and have also been associated with inflammatory bowel disease (IBD) and blood stream infections [3-7]. *Campylobacter* species are a leading cause of bacterial diarrhoeal disease worldwide, often linked to consumption of contaminated poultry [8-11]. *Campylobacter* infection can lead to Guillain–Barré syndrome [12,13]. Similarly, *Salmonella* species, especially *Salmonella enterica* serovars Typhimurium and Enteritidis, are responsible for a considerable proportion of food-borne illnesses [14].

Historically, bacterial culture has been the standard method for detecting enteric bacterial pathogens in diagnostic laboratories. However, in recent years, PCR methods have gained popularity for pathogen detection. Many countries have also adopted PCR methods for national surveillance of food-borne pathogens [15,16]. Therefore, evaluation of pathogen detection by bacterial culture and PCR is essential for accurately assessing the impact of transitioning from bacterial culture to PCR on detection rates in surveillance programmes. Although some previous studies compared the detection of *Campylobacter* and *Salmonella* species in faecal samples by bacterial culture and PCR, the sample sizes were small [17-19]. Comparative analysis of a larger number of clinical samples is needed to obtain more reliable and statistically robust information.

In the cited studies, *Aeromonas* species were most commonly cultured from specimens from individuals aged > 50 years and young adults, however, PCR method also detected a high infection rate in children aged < 2 years [2,20]. In the same studies, *Campylobacter* infections were more common among teenagers and young adults, but they were also a recognised cause of gastrointestinal infection in children. *Salmonella* gastrointestinal infections were reported frequently in young children [2,20]. *Aeromonas* blood stream infections were reported more commonly in older patients, with the gastrointestinal system being a common primary focus [21]. In patients aged > 65 years, gastrointestinal infections caused by *Campylobacter* and S*almonella* species were associated with more hospitalisations [22]. Despite these age-related differences in pathogen detection and clinical outcomes, the relationship between the pathogen load and patient age remains underexplored.

We aimed to compare the detection of *Aeromonas*, *Campylobacter* and *Salmonella* by concurrent bacterial culture and PCR using a large dataset and to analyse pathogen load across different age groups using quantification cycle (Cq) values as a surrogate marker. The findings from this study offer valuable insights for pathogen surveillance, laboratory diagnostics and clinical management.

## Methods

### Description of the dataset

We received a dataset from Douglass Hanly Moir (DHM) pathology laboratory in Sydney, Australia. In 2023, 90,291 faecal samples from patients with gastrointestinal infection symptoms (one sample from each patient) were tested for *Aeromonas*, *Campylobacter* and *Salmonella* species, by both bacterial culture and PCR.

Faecal samples included in this study were predominantly from community settings and submitted to DHM pathology by general practitioners and specialists such as gastroenterologists. The decision to request testing for gastrointestinal pathogens is made at the discretion of the treating clinicians, typically based on clinical symptoms. Testing for *Campylobacter* and *Salmonella* is routinely included in standard diagnostic panels for gastrointestinal infections across Australian states, as both pathogens are nationally notifiable. In contrast, *Aeromonas* is not currently part of the national surveillance system. However, in the DHM laboratory, stool samples are routinely tested for all three pathogens as potential causes for gastrointestinal infection.

### Culture methods

Cultivation and identification of enteric bacterial pathogens were conducted following the standard protocols in the DHM pathology laboratory. Faecal sample inoculation was performed using cotton swabs or pipettes for watery stool, with approximately the same amount of faecal material applied for each test.

Briefly, for the isolation of *Aeromonas* species, faecal samples were plated onto various agar plates, including xylose lysine deoxycholate (XLD) agar (characterised by pale yellow or yellow-pink flat colonies), thiosulfate citrate bile-salts sucrose (TCBS) agar (yellow or green flat colonies), cefsulodin-irgasan-novobiocin (CIN) agar (red with transparent border colonies) or horse blood agar (HBA) with an ampicillin (AMP) 25 μg disc (Oxoid, Basingstoke, the United Kingdom (UK)) and presumptive isolates were collected. Before inoculation onto TCBS plates, faecal materials were enriched in alkaline peptone water at 35°C for 6–8 h. Selective plates XLD and HBA were incubated at 35°C for 48 h, TCBS plates at 35°C for 24 h and CIN plates at 30°C for 48 h. All incubations were carried out under aerobic conditions.

Presumptive isolates were then tested for oxidase activity and oxidase-positive isolates were further identified using matrix-assisted laser desorption ionisation/time of flight mass spectrometry (MALDI/TOF-MS) (Vitek MS, bioMérieux, Marcy-l'Étoile, France). Confirmed isolates were reported as *Aeromonas* species, as MALDI/TOF-MS has limitations in reliably identifying *Aeromonas* species beyond the genus level [23,24].

For detection of *Campylobacter* species, faecal samples were plated onto *Campylobacter* Blood-Free Selective Agar (Thermo Fisher Scientific, Waltham, the United States (US)) and the plates were incubated at 42°C for 48 h under microaerophilic conditions. Suspected colonies were confirmed to the genus level based on positive catalase and oxidase tests, along with the characteristic gull-wing or curved morphology on Gram stain.

For detection of *Salmonella* species, all faecal samples were initially enriched in selenite broth at 35°C for 18–24 h. Following enrichment, two different selective plates were used. A loopful of the enriched broth was plated onto XLD plates and the plates were incubated at 35°C for 48 h under aerobic conditions, after which presumptive *Salmonella* colonies were confirmed to be lacking activity for pyroglutamyl aminopeptidase (PYR) and nitrophenylalanine deaminase (NPA) using the Oxoid Biochemical Identification System (OBIS) and further identified to the genus level using VITEK 2 GN Identification Card (bioMérieux). In parallel, a loopful of the enriched broth was plated onto *Salmonella* chromogenic agar plate and the inoculated plates were then incubated at 35°C for 18–24 h under aerobic conditions. Presumptive *Salmonella* isolates were further confirmed and identified as described above by OBIS and VITEK 2 GN Identification Card.

### PCR method

Faecal samples were tested with PCR without prior enrichment. Briefly, nucleic acids were extracted from 200 mg of stool or 200 μL of liquid stool using the Maelstrom 9600 instrument (TANBead, Taoyuan City, Taiwan). Extraction kits used were validated in-house as performing accurately before use for the Seegene assay (data not shown). Thereafter, the extracted DNA was tested for *Aeromonas*, *Campylobacter* and *Salmonella* by multiplex PCR using Seegene Allplex GI-Bacterial (I) Assay (Seegene, Seoul, South Korea) at the genus level. Samples with a Cq value ≤ 45 were considered positive if associated with an appropriate amplification curve according to the manufacturer’s instructions. Positive and negative controls in addition to a whole process internal control were run to assess for contamination and inhibition. As part of the validation study before accepting this assay in the DHM laboratory, precision studies to confirm the reproducibility and repeatability of this assay were performed and found to confirm the accuracy of the results obtained.

### Measurement of faecal calprotectin

Faecal calprotectin, a marker of intestinal inflammation, was measured using DiaSorin Liaison Chemiluminescent Immunoassay (CLIA, Saluggia, Italy) instrument with the same stool specimens that were tested for the bacterial pathogens.

### Statistical analyses

We calculated ratios of detection by Seegene PCR and bacterial culture for each of the three pathogens in faecal samples by age groups. The discrepancies between bacterial culture and PCR detection of *Aeromonas*, *Campylobacter* and *Salmonella* were also noted.

A logistic regression analysis was performed using a binomial generalised linear model to evaluate the relationship between patient age and sex with the detection rates of *Aeromonas*, *Campylobacter* and *Salmonella* species as described in previous studies [2,20].

To examine the relationship between Cq values with patient age and culture positivity, the median Cq value was regressed against patient age and culture positivity using quantile regression with the *quantreg package* [25]. The median was chosen because diagnostic residual plots from linear regression of Cq values, along with several transformations of Cq values, revealed distributional assumption violations (Cq values were not normally distributed), which could affect the reliability of linear regression [26]. To estimate the relationship between Cq values and levels of faecal calprotectin, a left-censored regression model was used for *Aeromonas* and *Campylobacter*, as levels of faecal calprotectin were censored for values < 5 μg/g [27]. For *Salmonella*, with no censoring, a linear model was used. In all three cases, faecal calprotectin was log transformed. Wald tests for the slope between Cq values and faecal calprotectin were used to determine evidence of a trend.

All statistical analyses were performed using R software (version 4.5), along with R Studio (https://www.r-project.org) and p < 0.05 or 95% confidence intervals (CI) not overlapping zero were taken to indicate evidence for each effect.

## Results

### Detection of *Aeromonas*, *Campylobacter* and *Salmonella* species

Of the 90,291 faecal samples, 480 (0.5%) were positive for *Aeromonas* by culture and 2,651 (2.9%) by Seegene PCR (Table 1). The ratio of detection by Seegene PCR and culture was 5.5. For *Campylobacter*, the corresponding numbers were 2,807 (3.1%) by culture and 3,773 (4.2%) by PCR, respectively. The ratio of detection by PCR and bacterial culture was 1.3. For *Salmonella*, 745 (0.8%) samples were positive by culture (*S.* Typhi was identified in five faecal samples and the remaining 740 were non-typhoidal *Salmonella* species) and 669 (0.7%) were positive by PCR. The ratio of detection by PCR and bacterial culture was 0.9.

**Table 1:** Detection of *Aeromonas*, *Campylobacter* and *Salmonella* by bacterial culture and PCR^a^ in faecal samples from patients with gastrointestinal symptoms, Sydney, Australia, 2023 (n = 90,291)

Bacterial genus	Culture		PCR		PCR/culture
	Detection	%	Detection	%	
*Aeromonas*	480	0.5	2,651	2.9	5.5
*Campylobacter*	2,807	3.1	3,773	4.2	1.3
*Salmonella*	745	0.8	669	0.7	0.9

^a^ PCR tested using a commercial Seegene multiplex PCR assay. For *Salmonella* species culture, faecal samples were enriched in selenite broth.

Co-infections by more than one of these pathogens were observed. By culture, *Aeromonas* and *Campylobacter* were detected in 53 samples, *Aeromonas* and *Salmonella* in 17, *Campylobacter* and *Salmonella* in 33. No samples were positive for all three pathogens. By PCR, the corresponding numbers were 195, 39, 29 and 2, respectively. When a sample tested positive for more than one pathogen, it was counted positive for each of these pathogens.

### Discrepancies between culture and Seegene PCR

Of the 480 faecal samples positive for *Aeromonas* by culture, 92 (19.2%) were negative by Seegene PCR; of the 2,807 samples positive for *Campylobacter* by culture, 102 (3.6%) were negative by PCR and of the 745 samples positive for *Salmonella* by culture, 171 (23.0%) were negative by PCR. Of the 2,651 samples positive for *Aeromonas* by PCR, 2,170 (81.9%) were negative by culture. Of the 3,773 samples positive for *Campylobacter* by PCR, 966 (25.6%) were negative by culture. Of the 669 samples positive for *Salmonella* by PCR, 95 (14.2%) were negative by culture.

### Detection by age and sex

**Figure 1:**
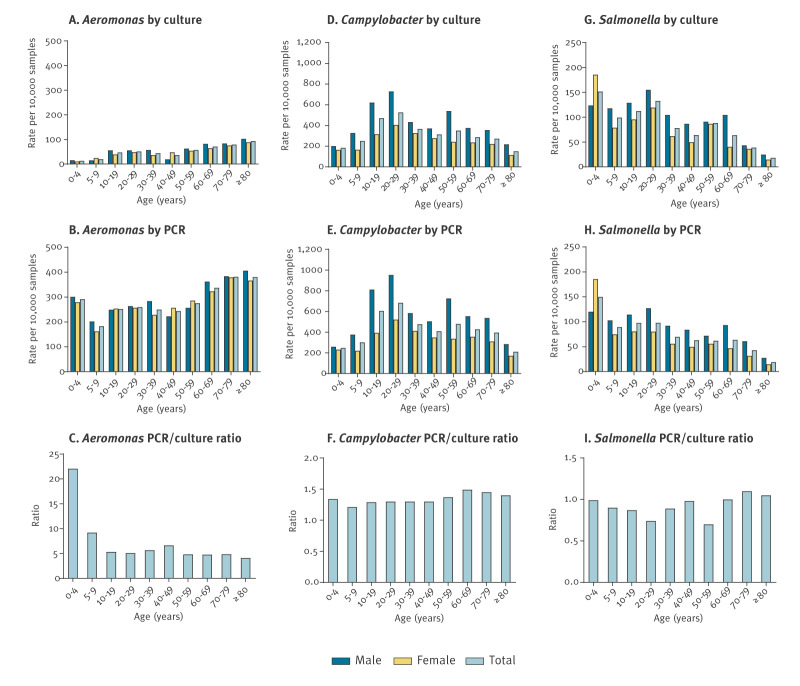
Detection of *Aeromonas*, *Campylobacter* and *Salmonella* species by culture and PCR^a^, stratified by age and sex, in faecal samples from patients with gastrointestinal symptoms, Sydney, Australia, 2023 (n = 90,222)

^a^ A commercial Seegene multiplex PCR assay.

Detection by age and sex using culture and Seegene PCR are shown in Figure 1 and Table 2. There were 69 patients without information on sex, and they were excluded from this part of the analysis. Among children and adolescents, detection of *Aeromonas* was most common in samples from 0–4-year-olds (309/10,610; 2.9%) by PCR, but not by culture (14/10,610; 0.1%) (Figure 1A–B, Table 2). With PCR, *Aeromonas* was detected in 2.6% (223/5,361) of samples from young adults aged 20–29 years. Detection was most common in samples from patients aged > 60 years. More samples tested positive for *Aeromonas* by PCR, with the highest PCR/culture ratio in children aged 0–4 years (ratio 22.1) (Figure 1C). Irrespective of the method, the detection rates were significantly associated with patient age (p < 0.001 for both methods) but not with sex (Table 3). Increasing patient age was associated with a greater odds ratio (OR) of detection of *Aeromonas*.

**Table 2:** Detection of *Aeromonas*, *Campylobacter* and *Salmonella* species by culture and PCR^a^, stratified by age and sex, in faecal samples from patients with gastrointestinal symptoms, Sydney, Australia, 2023 (n = 90,222)

Age (years)	*Aeromonas*								*Campylobacter*								*Salmonella*								Samples tested (n)		
	Culture				PCR				Culture				PCR				Culture				PCR				Female	Male	Total
	Female	Male	Total	%	Female	Male	Total	%	Female	Male	Total	%	Female	Male	Total	%	Female	Male	Total	%	Female	Male	Total	%			
0–4	5	9	14	0.1	134	175	309	2.9	79	117	196	1.8	111	151	262	2.5	89	72	161	1.5	89	70	159	1.5	4,795	5,815	10,610
5–9	6	4	10	0.2	39	53	92	1.8	40	86	126	2.5	53	99	152	3.0	19	31	50	1.0	18	27	45	0.9	2,406	2,627	5,033
10–19	13	19	32	0.5	85	85	170	2.5	106	212	318	4.7	132	277	409	6.1	32	44	76	1.1	27	39	66	1.0	3,352	3,408	6,760
20–29	26	18	44	0.5	138	85	223	2.6	217	235	452	5.3	280	307	587	6.8	64	50	114	1.3	43	41	84	1.0	5,361	3,221	8,582
30–39	24	23	47	0.4	151	114	265	2.5	216	174	390	3.7	272	235	507	4.8	41	42	83	0.8	37	37	74	0.7	6,599	4,018	10,617
40–49	28	7	35	0.4	150	82	232	2.4	162	137	299	3.1	204	186	390	4.1	29	32	61	0.6	29	31	60	0.6	5,839	3,683	9,522
50–59	30	20	50	0.6	158	82	240	2.8	134	172	306	3.5	186	232	418	4.8	48	29	77	0.9	31	23	54	0.6	5,527	3,192	8,719
60–69	40	29	69	0.7	200	128	328	3.4	146	133	279	2.9	220	196	416	4.3	25	37	62	0.6	29	33	62	0.6	6,187	3,532	9,719
70–79	48	33	81	0.8	240	151	391	3.8	140	140	280	2.7	196	211	407	4.0	23	17	40	0.4	20	24	44	0.4	6,325	3,928	10,253
≥ 80	60	37	97	0.9	249	147	396	3.8	78	79	157	1.5	117	103	220	2.1	10	9	19	0.2	10	10	20	0.2	6,790	3,617	10,407

^a^ A commercial Seegene multiplex PCR assay.

**Table 3:** Association of age and sex with detection of *Aeromonas*, *Campylobacter* and *Salmonella* species by culture and PCR^a^ in faecal samples from patients with gastrointestinal symptoms, Sydney, Australia, 2023 (n = 90,222)

Bacterial genus		p value	OR	95% CI
*Aeromonas*				
Culture	Age	< 0.001	1.016	1.013–1.020
	Sex	0.222	1.121	0.932–1.345
PCR	Age	< 0.001	1.006	1.004–1.007
	Sex	0.124	1.064	0.983–1.151
*Campylobacter*				
Culture	Age	< 0.001	0.997	0.995–0.998
	Sex	< 0.001	1.606	1.489–1.733
PCR	Age	< 0.001	0.998	0.997–0.999
	Sex	< 0.001	1.632	1.528–1.743
*Salmonella*				
Culture	Age	< 0.001	0.983	0.980–0.986
	Sex	0.007	1.221	1.055–1.413
PCR	Age	< 0.001	0.984	0.981–0.987
	Sex	0.001	1.292	1.108–1.507

CI: confidence interval; OR: odds ratio.

^a^ A commercial Seegene multiplex PCR assay.

The relationships between detection rates of these three pathogens by both bacterial culture and PCR were analysed by logistic regression using a binomial generalised linear model. The OR for age represents the change in odds of detection per additional year older in age. The OR for sex represents the ratio of the odds of detection comparing males with females (reference group).

Detection of *Campylobacter* was most common in young adults aged 20–29 years (culture: 452/8,582; 5.3%; PCR: 587/8,582; 6.8%) (Figure 1D and 1E, Table 2). In all age groups, more samples were positive with PCR than with culture (PCR/culture: 1.2–1.5) (Figure 1F). Detection was significantly associated with patient age (p < 0.001 for both methods) and sex (p < 0.001 for both methods) (Table 3), with the odds of detection decreasing for older patients and increasing in male patients.

*Salmonella* infection was most common in children aged 0–4 years, as detected by culture (161/10,610; 1.5%) and PCR (159/10,610; 1.5%) (Figure 1G and 1H, Table 2). By culture, *Salmonella* was detected in 114 (1.3%) of 8,582 samples from young adults aged 20–29 years, however, this was not apparent by PCR (84/8,582; 1.0%). Compared with culture method, fewer samples were positive with PCR in persons aged < 60 years (culture: 622/59,843; PCR: 542/59,843) (Figure 1I, Table 2). In patients aged ≥ 60 years, the bacterial culture detection ratios were 1.00–1.10 (Figure 1I). With both methods, detection was significantly associated with patient age (p < 0.001 for both methods) and sex (p = 0.007 for culture and p = 0.001 for PCR method) (Table 3).

### Associations between quantification cycle (Cq) values and patient age, detection by culture and faecal calprotectin

**Figure 2:**
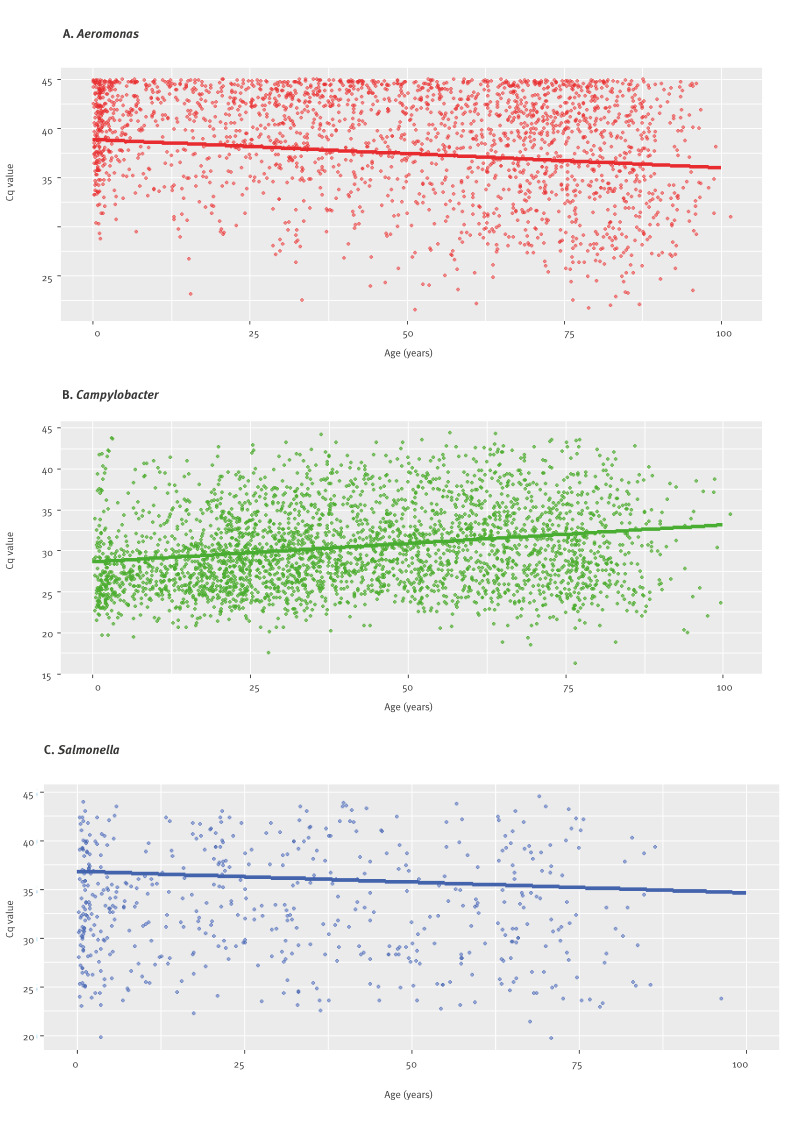
Association between quantification cycle (Cq) values of PCR^a^ and patient age in detection of *Aeromonas* and *Campylobacter* species in faecal samples from patients with gastrointestinal symptoms, Sydney, Australia, 2023 (n = 90,291)

^a^ A commercial Seegene multiplex PCR assay.

For *Aeromonas*, there was a significant association between patient age and Cq values (p < 0.001) with a negative trend (slope = −0.03; 95% CI: −0.04 to −0.02). For *Campylobacter,* a significant association between patient age and Cq values was also observed (p < 0.001). However, unlike the negative trend in *Aeromonas* infections, the trend was positive for *Campylobacter* (slope = 0.05; 95% CI: 0.04 to 0.05). For *Salmonella,* no statistically significant association between patient age and Cq values was seen (Figure 2 and Table 4).

Panel A. A significant association between Cq values and patient age (p < 0.001) was observed, with a negative trend (slope = −0.03; 95% CI: −0.04 to −0.02).

Panel B. A significant association between Cq values and patient age (p < 0.001), but with a positive trend (slope = 0.05; 95% CI: 0.04 to 0.05).

Panel C. No significant association between Cq values and patient age was observed*.*

**Table 4:** Regression analysis of PCR^a^ quantification (Cq) values for detection of *Aeromonas*, *Campylobacter* and *Salmonella* species in faecal samples from patients with gastrointestinal symptoms by age and culture positivity, Sydney, Australia, 2023 (n = 90,291)

Characteristic	Coefficient	SE	df	Lower CL	Upper CL	t value	p value
*Aeromonas*							
Intercept	41.938	0.244	2,290	41.459	42.416	171.935	< 0.001
Age	−0.029	0.005	2,290	−0.039	−0.019	−5.764	< 0.001
Detection by culture	−6.123	0.457	2,290	−7.019	−5.227	−13.398	< 0.001
*Campylobacter*							
Intercept	30.259	0.282	3,168	29.706	30.812	107.266	< 0.001
Age	0.045	0.004	3,168	0.037	0.054	10.268	< 0.001
Detection by culture	−3.286	0.262	3,168	−3.799	−2.772	−12.544	< 0.001
*Salmonella*							
Intercept	39.350	1.066	529	37.257	41.444	36.922	< 0.001
Age	−0.014	0.014	529	−0.041	0.014	−0.964	0.335
Detection by culture	−6.557	1.056	529	−8.632	−4.482	−6.209	< 0.001

CL: confidence level; df: degrees of freedom; SE: Standard error of coefficient.

^a^ A commercial Seegene multiplex PCR assay.

The coefficient for age gives the estimated change in median Cq values for an increase of 1 year, while the coefficient for culture positivity represents the difference between culture positive and culture negative median Cq values. Uncertainty is quantified by the lower and upper limits of the 95% confidence interval (lower CL and upper CL respectively), which show a range of plausible values of this coefficient. p values are for Wald tests of each coefficient being different to zero. Detection by culture was controlled for in the quantile regression which estimated these coefficients.

For all three pathogens, the association between lower Cq values and detection by culture was significant (*Aeromonas*: difference = −6.12; p < 0.001; *Campylobacter*: difference = −3.29; p < 0.001 and *Salmonella*: difference = −6.56; p < 0.001) (Table 4).

A subset of faecal samples had faecal calprotectin tested under the request of referring clinicians. After excluding patients diagnosed with IBD or with infections with more than one pathogen, 245 *Aeromonas-*positive faecal samples, 146 *Campylobacter*-positive faecal samples and 22 *Salmonella*-positive faecal samples could be included in the analysis. A significant negative relationship between Cq values and levels of faecal calprotectin was found for *Aeromonas* (slope = −0.17; p < 0.001), *Campylobacter* (slope = −0.12; p < 0.001) and *Salmonella* (slope = −0.15; p = 0.02).

## Discussion

We analysed a large dataset of concurrent testing of faecal samples for *Aeromonas*, *Campylobacter* and *Salmonella* by culture and Seegene PCR. Compared with culture, more samples were positive by PCR for *Aeromonas* and *Campylobacter* (5.5-fold and 1.3-fold, respectively), but fewer for *Salmonella* species (0.9-fold). *Campylobacter* and *Salmonella* species have been included in surveillance programmes of food-borne pathogens in many countries, and these programmes have increasingly adopted PCR methods in recent years. In our study, Seegene PCR increased the detection of *Campylobacter* species by 34.4% and decreased the detection of *Salmonella* species by 10.2% compared with bacterial culture. This impact on pathogen detection due to change of detection method should be considered when assessing the infection rates of these pathogens in surveillance programmes.

While Seegene PCR assay increased the detection of *Campylobacter* and *Aeromonas* compared with culture, some limitations must be considered when interpreting results. Firstly, PCR detects bacterial DNA from both viable and dead cells, meaning that a positive PCR result does not necessarily indicate an active infection. Secondly, the Seegene PCR we used identifies pathogens at the genus level, which may include *Campylobacter* species that are not culturable under standard culture methods optimised for *C. jejuni* and *C. coli* or are less pathogenic or non-pathogenic.

We observed a substantial proportion of culture-positive faecal samples that were negative for *Aeromonas*, *Campylobacter*, *Salmonella* by Seegene PCR*,* with the highest proportion observed for *Salmonella* (23.0%). This may reflect the presence of PCR inhibitors in faecal samples, which can vary greatly between individuals [28]. Each method has its inherent limitations, and it is difficult to judge one test superior to the other when results are discordant. Combining both methods improves detection of these pathogens. These findings support the use of both methods to improve detection; however, the additional cost and resource requirements must be considered in routine practice. In some clinical scenarios, such as the evaluation of patients with suspected IBD, using both methods may be justified to help exclude infectious causes of gastrointestinal inflammation. In this study, only one faecal sample per patient was used for bacterial culture; collecting multiple faecal samples may increase detection [29].

In most age groups, fewer samples were positive by PCR for *Salmonella* species compared with culture. Prior to culture, faecal samples were enriched in selenite broth, thereby enhancing pathogen recovery, whereas samples analysed by Seegene PCR were not enriched. Similarly, using enrichment or selective methods before plating may improve sensitivity of culture methods for detection of *Aeromonas*.

*Aeromonas* was more often detected in samples from young children, young adults and individuals aged > 50 years. *Campylobacter* was most common in adolescents and young adults aged 10–29 years, while *Salmonella* infections were more common in children < 4 years. Furthermore, *Campylobacter* and *Salmonella* were significantly more often detected in samples from males, as in our previous study using data from 2015–2019 [20]. In that dataset, detection of *Aeromonas* was associated with male patients, contrary to the current study, suggesting an increase in *Aeromonas* infections in females more recently.

Quantification values in PCR assays are inversely proportional to the amount of target nucleic acids with lower Cq values reflecting higher amounts of target nucleic acids in the sample. In our study, we used Cq values as a surrogate of pathogen load. Unexpectedly, we noticed a contrasting relationship between Cq values and patient age for *Aeromonas* and *Campylobacter*. The Cq values were negatively associated with patient age for *Aeromonas,* meaning that older patients had higher pathogen loads, while for *Campylobacter*, Cq values were positively associated with patient age, indicating lower pathogen load in older patients. Using Cq values as an indicator of pathogen load has limitations, as Cq values can be influenced by multiple factors such as sample quality, nucleic acid extraction efficiency, and presence of PCR inhibitors. While these factors can introduce uncertainty, the associations we observed between Cq values and culture positivity as well as faecal calprotectin support that Cq values carry biologically and clinically relevant information as a surrogate for pathogen load in this context. The pathogenicity of *Aeromonas* species is supported by the similar significant negative relationship between Cq values and levels of faecal calprotectin as found for the more established pathogens, *Campylobacter* and *Salmonella* species. However, faecal calprotectin was measured in only a subset of samples, and further studies including larger numbers of stool specimen and additional inflammatory markers are needed to confirm the relationship between pathogen Cq values and intestinal inflammation.

White et al. (2019) previously reported a significant negative correlation between clinical symptoms and patient age in *Campylobacter* gastroenteritis [22]. Our finding of higher Cq values for samples positive for *Campylobacter* in older adults could partly explain their observation of fewer clinical symptoms in this age group [22]. The increased hospitalisation rate in adults aged > 60 years with *Campylobacter* infection, as noted by White et al. (2019), is likely attributable to host factors, such as age-related immunity decline and underlying health conditions.

The significantly higher *Aeromonas* bacterial load in older adults is a notable health concern. In vitro studies have shown that increased *Aeromonas* bacterial load leads to more inflammation and damage to human intestinal epithelial cells and macrophages [7,30]. *Aeromonas* gastrointestinal infections are more likely to progress to bloodstream infections in patients aged > 60 years, with mortality rates from 39.1% to 66.8% [31,32]. Our findings support *Aeromonas* species could be important enteric pathogens in older adults and may lead to severe disease. Early antimicrobial treatment for *Aeromonas* gastrointestinal infections in older adults may decrease the severity of illness and reduce disseminated infection.

The reasons why older adults had higher *Aeromonas* pathogen load in gastrointestinal infections are not fully understood but could be related to immunosenescence or underlying co-morbidities. Additionally, older individuals may be more frequently exposed to *Aeromonas*-contaminated water or foods by having lower levels of hygiene. Identifying the primary sources of human gastrointestinal infections is therefore critical in reducing these infections.

The Seegene PCR method detected an infection peak of *Aeromonas* in young children aged 0–4 years, which was not detected by culture. The negative correlation between Cq values and patient age suggests that children in this age group have lower *Aeromonas* pathogen load, which is consistent with the fewer detections by culture.

## Conclusion

In conclusion, in this large community-based study, we showed that detection of gastrointestinal pathogens differed substantially between culture and PCR and varied by pathogen. With PCR, we detected more *Aeromonas* and *Campylobacter* but fewer *Salmonella* than with culture. For *Aeromonas* and *Campylobacter*, pathogen loads showed opposite associations with age. Higher pathogen loads were associated with increased culture positivity and faecal calprotectin levels across pathogens. These findings highlight the importance of pathogen- and method-specific interpretation of PCR and culture results in diagnostic testing and surveillance.

## Data Availability

Under the current ethics approval, clinical data used in this study are not publicly available.
